# Evaluation of experiences of the patients discharged from the COVID-19 intensive care unit: a qualitative research

**DOI:** 10.1038/s41598-023-46818-1

**Published:** 2023-11-10

**Authors:** Serap Torun, Esra Bulmuş, Osman Bilgin

**Affiliations:** 1 Department of Nursing Administration, Health Science Faculty, Çukurova University, Adana, Turkey; 2 Adana City Training and Research Hospital, Adana, Turkey

**Keywords:** Health care, Diseases, Infectious diseases

## Abstract

Making arrangements by learning how intensive care patients feel due to a disease called as fatal worldwide can make it easier for patients to cope with the disease. For this reason, it is important for healthcare professionals to understand the patients who have been infected and discharged during the COVID-19 pandemic. The experiences of the patients may affect the perspective of the disease and cause different changes in the perception of it. This study, which was conducted based on this idea, aimed to examine the intensive care experiences of patients discharged from the COVID-19 intensive care unit. This study used a phenomenological qualitative approach. A semi-structured interview form was used to interview 23 patients discharged from the COVID-19 intensive care unit. The findings were reported on the basis of consolidated criteria for reporting qualitative research. In line with the data obtained from the interviews, five main themes and eight subthemes were created. The main themes were classified as emotional expressions (positive/negative) related to intensive care experience, coping methods, analogies (for COVID-19 and nurses) and attitudes towards the care provided (respiration, nutrition, excretion and privacy, sleep, communication). In this study, the participants experienced negative emotions such as fear of death/anxiety, sadness, loneliness, and helplessness during their intensive care experiences. Most of them stated that they tried to cope with prayer and communication. The participants compared COVID-19with deadly and respiratory-inhibiting tools and diseases. They expressed difficulties in breathing, nutrition, excretion and privacy, sleep and communication related to the care provided. In this process, they made positive analogies for the nurses who spent the most time with them, such as angels and family members.

The World Health Organization (WHO) considered COVID-19, which affected 210 countries, to be a public health problem by describing it as a pandemic^1,2^. While COVID-19 was spreading at an extraordinary rate, it caused severe panic and anxiety in humans with a high morbidity and mortality rate^2^. To correct this, public policies such as social distancing, isolation and self-quarantine have been implemented worldwide. These compulsory changes in daily life have created psychological effects such as deep fear/anxiety in individuals^3^. Arrangements have been made for manpower and physical environments in the health sector to prevent the worldwide spread of the disease and to provide treatment and control^4^. Apart from preventive measures, arrangements were made to care for patients diagnosed with COVID-19 in Turkey, the bed capacities of hospitals were increased, all elective surgeries were postponed, and the distribution of nurses throughout the hospital was regulated. Some units have been arranged to serve only COVID-19 patients, and additional intensive care units have been opened^5^.

Intensive care units are clinics where more advanced technology is used, intensive drug therapy as well as close monitoring and care practices are applied in order for individuals with impaired vital functions to maintain their normal vital functions^6^. COVID-19 has heterogeneous clinical symptoms such as fever, cough, dyspnea, muscle pain and fatigue. Patients, who develop acute kidney injury and multiple organ failure, especially severe acute respiratory failure due to pulmonary involvement in COVID-19 patients, should be admitted to the intensive care unit^7^.

COVID-19 causes psychosocial problems besides physiological disorders in patients. According to the literature, as the length of stay in the intensive care unit increases, patients experience severe psychosocial health problems, posttraumatic stress disorder, depression, anxiety disorders and cognitive disorders^8–10^. The risk of transmission of the disease has affected the attitudes of healthcare professionals towards patients. The use of protective equipment has caused patients to have communication problems. Patients neither could not see the face of the person providing health care nor could make a professional distinction. Due to the pandemic, compulsory professional regulations have also been a factor in anxiety and stress in nurses. This is thought to affect patient care and therefore patient outcomes^11^.

Making arrangements by learning how intensive care patients feel due to a disease called as fatal worldwide can make it easier for patients to cope with the disease. For this reason, it is important for healthcare professionals to understand the patients who have been infected and discharged during the COVID-19 pandemic. The experiences of the patients may affect the perspective of the disease and cause different changes in the perception of it. As a matter of fact, studies have shown that Covid-19 causes patients to experience feelings such as "pain, exhaustion, hopelessness, helplessness, fear of death"^12,13^. This study, which was conducted based on this idea, aimed to examine the intensive care experiences of patients discharged from the COVID-19 intensive care unit.

## Materials and methods

### Research model

This is a qualitative phenomenological study.

### Research sample

The study was conducted in the COVID-19 intensive care unit of a City Training and Research Hospital in Turkey. The population of the study consisted of 159 patients discharged from the COVID-19 Intensive Care Unit. Purposive sampling was used in the research. The study included patients who were discharged from the COVID-19 intensive care unit between March 1, 2021 and July 31, 2022 and who agreed to participate in the study. Data saturation was dynamically determined by analysis after each interview and the study was terminated as sufficient data saturation was achieved after the 23 interviews.

### Research team

All of the investigators were RNs. A researcher who holds the title of associate professor in the field of nursing has qualitative and quantitative studies on the ethical dimensions of patient cases. The other researcher, who is continuing her doctoral education in the field of nursing, has received training and conducted studies on qualitative studies. The other researcher, who is in direct contact with the patients, has been a nurse in the intensive care unit for many years.

### Data collection

The research data were collected by the all researchers between May 20 and August 31, 2022. After the patients were discharged from the intensive care unit, an average of 6 months passed until the interview report was written. While collecting the research data, twelve questions, including the sociodemographic characteristics created by the researchers and five questions in the semi-structured information form, were transferred to the interviewees, and their answers/statements were recorded by voice recording in the interview. After the preliminary information, the participants who wanted to participate were interviewed on the phone and one participant was interviewed face-to-face upon his request and the interview was recorded. At the beginning of the interview, the researcher informed the participants that their interview would be recorded and used only to collect data in the research. The participants were informed that they could have a break during the interview or withdraw from it at any time. After reading the prepared informed consent text, asking whether they wanted to continue the interview or not, and obtaining their consent, audio recording was taken. No one took part in the interviews except for the researcher and interviewer. The average duration of the interview with each participant was 20 min. To examine how patients discharged from the COVID-19 intensive care unit evaluated their intensive care experience, answers to the following questions were sought:

- Question 1: What feelings did you have during your stay in the COVID-19 intensive care unit?
- Question 2: How did you cope with the situations and feelings that worried/frightened you in the COVID-19 intensive care unit?
- Question 3: If you were to compare COVID-19 with an object, what would this object be?
- Question 4: What would you like to say about the care provided to you in the COVID-19 intensive care unit?
- Question 5: If you were to describe the nurses who cared for you during this process, what would you liken to them?

### Data analysis

After the interviews with the participants were completed, the audio recordings were transferred to the computer in written text by the researchers. The inductive thematic analysis method was applied in the analysis of the data. Each interview text was read by three researchers and content analysis was conducted. Thematic coding was done by coding each statement that was deemed meaningful. The main themes and subthemes were determined with the common opinion of the researchers. While transferring qualitative data, reliability was increased by quoting the interviews one-to-one. P1-P23 codes were used for the interviewees.

### Research ethics

To conduct the research, necessary permissions were obtained from the Ethics Committee of Non-Interventional Clinical Research (Decision no.122/53 dated May 22, 2022), the Provincial Health Directorate and the hospital. Verbal informed consent was obtained from the participants. The study was conducted in accordance with the principles of the Declaration of Helsinki.

## Results

In the study, the mean age of the participants discharged from the COVID-19 intensive care unit was 48.86 (min:30, max:65), and 13 of them were male. The duration of the participants’ stay in the intensive care unit varied between two and 49 days. Twelve of the participants were literate and two had an undergraduate degree. Eight patients worked, 15lived in the province and three lived in the village. Eleven patients stated that they had a chronic disease. Seven participants could not see their relatives during their hospitalization in the intensive care unit, while the others had the opportunity to see their relatives from a distance or from the glass partition by taking protective measures. Seven participants did not see their relatives during their stay in the intensive care unit (Table 1). The codes, main themes and subthemes obtained from the participants’ expressions regarding their experiences in the intensive care process are given in Table 2.

**Table 1:** Distribution of sociodemographic characteristics of the participants discharged from the intensive care unit (n = 23).

	Age	Sex	Marital status	Chronic diseases	Length of hospital stay (days)	Interviewing relatives
P1	59	Female	Married	No	18	Yes
P2	65	Male	Married	No	4	Yes
P3	57	Male	Married	No	19	Yes
P4	61	Male	Married	No	2	No
P5	51	Female	Single	No	14	No
P6	51	Male	Married	No	9	Yes
P7	64	Male	Married	Hypertension	20	Yes
P8	59	Male	Married	Chronic renal failure, heart, diabetes, hypertension	10	No
P9	30	Male	Married	No	3	Yes
P10	34	Female	Married	No	5	Yes
P11	45	Female	Married	No	30	Yes
P12	38	Female	Married	No	1	Yes
P13	31	Female	Married	Asthma, diabetes	20	Yes
P14	60	Male	Married	Heart, diabetes	15	Yes
P15	62	Male	Married	Diabetes	49	Yes
P16	32	Female	Married	Hypertension, chronic renal failure, heart failure	20	No
P17	42	Male	Married	Diabetes	4	Yes
P18	51	Female	Married	Chronic renal failure	14	No
P19	35	Male	Married	No	14	No
P20	55	Female	Married	Diabetes, panic attack, hypertension	2	No
P21	61	Male	Married	Rectum cancer	5	Yes
P22	48	Male	Married	Chronic renal failure, hypertension	9	Yes
P23	33	Female	Married	No	10	Yes

**Table 2:** Themes, subthemes and codes created with the expressions of the participants (n = 23).

Themes	Codes	n
Theme 1: Emotional expressions regarding the COVID-19 intensive care experience		
Positive statements	Nurses' interest and good behaviors	19
	Receiving news from the family	9
Negative statements	Respiratory distress	17
	Fear of death	16
	Nutritional distress	13
	Concerns about their families	10
	Insomnia	8
	Sounds in the intensive care unit	5
	Invasive interventions	5
	Loneliness	4
	Pain	4
Theme 2: Coping methods		
	Faith in Allah and prayer	17
	Nurses' support	14
	The thought of reuniting with the family	8
	Person who cannot cope	2
Theme 3: Simulations for COVID-19 disease		
	Death	5
	Flu	4
	Monster	3
	Haul	2
	It cannot be compared to anything	2
	Microbe	1
	Stopper in the lung	1
	The wolf inside me	1
	Hose	1
	Bag in the head	1
	Enemy	1
	Something very bad	1
Theme 4: Experiences with care offered		
Respiration	Respiratory distress	17
Nutrition	Nutritional distress (loss of appetite, inability to eat orally, inability to reach food)	13
Discharge and privacy	Shame-hesitation	12
	Unclothed	11
	Usage of diapers	9
Sleep	Insomnia	8
	Invasive interventions	5
Contact	Receiving news from the family	9
	Loneliness	4
Theme 5: Simulations for the nursing profession		
	Angel	13
	A family member	3
	Flower	2
	Beings in hell	2
	Star	1
	Ladybird	1
	Sergeant	1

### Theme 1. Emotional expressions regarding the COVID-19 intensive care experience

This theme is divided into two subthemes: positive and negative expressions. Four interviewees provided positive expressions about the intensive care experience. Most interviewees stated that they experienced negative feelings and had concerns. Twelve participants stated that they had a direct fear of death. Three interviewees, who had been pregnant during the disease and had just given birth, expressed their concerns about their babies. Two interviewees stated that they were afraid, but staying in the hospital for a long time was the reason for fear. Examples of the participants’ statements are given in Table 3 and there are examples of statements that are excluded in the table below (Table 3).

**Table 3:** Examples from the participants’ expressions according to themes and subthemes (n = 23).

Theme 1.Emotional expressions regarding the COVID-19 intensive care experience
Subtheme 1.1: Positive statements - “It felt good because my breath was at normal levels, my oxygen was around90s, and I got rid of the oxygen stuck in my nose…” (P10) - “My brother came in. Imagine you're abroad and you see a fellow citizen, and he becomes a brother to you. You do not see anyone, you get one visitor all day long. How exciting it is…” (P17)
Subtheme 1.2 Negative statements - “I felt very lonely at that moment. If I die, no one comes to my funeral. The funeral prayers of the dead are not performed. It hurts to go on that last journey like that…” (P7) - “I do not have 30 days in my memory. I was hallucinating for 30 days. I do not remember my 30–35 days at all. I dealt with hallucinations…” (P15) - “Is it fifty percent whether you may get out? It is depressing. You follow your heartbeat. You say that we will never get out of here when it gets higher. They packed and took away those who died in a gray box…” P22
Theme 2. Coping methods
- “I prayed, I did not panic. Nurses were my best hope and were next to me 24/7. If I do not get out of here alive, it is the will of Allah…” (P7) - “I hoped to get my daughter back. I think I was able to stay strong because of my sense of motherhood. The support of my husband and the staff has been good. I prayed hard.” (P13)
Theme 3.Analogies for COVID19
That is terrible. Monster, an invisible distress, filth…” (P2) - “COVID-19,the stopper in the lung…” (P3) - “Like Azrael…” P22 - “Put a bag in your head and try to breathe. This is the only way you will understand me. Like a bag on your head…”P23
Theme 4. Experiences with care provided
Subtheme 4.1 Respiration - “I was having difficulty breathing. I was so afraid that I would die from the last breath…” (P16) “If your arm or leg were to be severed, you would only suffer from its local pain, but it is extraordinary not to be able to breathe….”P23 Subtheme 4.2 Nutrition - “I knew I had to eat. It took me an hour to eat. I had a fear. I avoided drowning due to something in my throat and a bite in my mouth. It took me an hour to get ready to eat…” (P3) - “The pipe you eat from is an issue, the pipe you breathe from is another. I know too, but while eating, a sputum comes, the filter of the tracheostomy gets clogged…” (P15) Subtheme 4.3 Discharge and privacy - “First, they took me to bed. They took my top off…” (P6) - “Sometimes I was so embarrassed when they were cleaning my diapers. It was too much for me. I was ashamed of the nurses…” (P7) - “I cannot soil my pants if they kill me. Even if I am dying, I have to go to the restroom. I did not agree to use diaper…” (P16) Subtheme 4.4 Sleep - “I did not get any sleep until morning. I'm not sleepy. I could not sleep because of the pain in my head and body… " (P4) - “There is something that nags at you. How are you going to spend your time? What would happen? I cannot sleep a wink. I did not sleep there for a second…” (P20) Subtheme 4.5 Communication - “Nurses and doctors showed a lot of interest. I have seen a lot of attention. The nurses treated me properly…” (P4) - “I have not heard from my family. My wife, my twins, my baby, did they make it? We understand that the phone is forbidden, but this prohibition has to be canceled some how. Maybe I will die. What is the matter? I may want to see my family for the last time. Let me see them once. They could say no…”P22
Theme 5. Analogies for nurses
“I liken nurses to the sergeants of my time in the army. They took a slave there. They arose the feeling what kind of people do we make live…” (P17) “Everyone showed much interest in me. They did a lot more than nursing. They were like angels on my shoulders all the time. It felt safe that they quickly came and followed…”P23

#### Subtheme 1.1 Positive statements

“At that moment, I felt that I would be saved. I felt no fear because of the compassion that nurses and caregivers showed me…” (P3).

“The support of my husband, doctors and nurses was good…” (P13)

#### Subtheme 1.2 Negative statements

I was scared, I was so scared. I felt guilty, emptiness. I was afraid that I would stay for a long time…" (P10)

“I was expecting twins. My pregnancy made the fear that something would happen to me and my babies. I felt my muscles melting. Even if I did not lose my babies, the X-rays and the drugs I was taking made me worried that it would hurt them. Their motions made me feel as if they were saying, “We are hungry, mom…”(P23).

### Theme 2. Coping methods

The participants were asked about how they coped with the situations and emotions that worried/frightened them during the intensive care process. They stated that they coped with the negative emotions they experienced in the intensive care by constantly praying, with the support of healthcare professionals, the wish to meet their families and listening to the radio. Two participants reported that they could not cope with the negative emotions they experienced during their stay in the intensive care (Table 3). Some of the participants’ statements are as follows:

“I dealt with it by taking refuge in Allah most. You are overcome everything with Him…" (P18)

“I met my family for 10–15 s. That is how I handled it. Otherwise, I would have gone mad…” (P22)

### Theme 3. Analogies

This theme is divided into two sub-themes: "Analogies for Covid-19" and "Analogies for nurses". The meanings attributed to beings, objects and situations and the feelings felt toward them can be expressed by symbolization or analogy.

#### Subtheme 3.1. Analogies for Covid-19

For COVID-19, participants made analogies such as "death", "flu", and "monster". Two participants could not compare the disease to anything and one participant expressed it *as* "a very bad thing" (Table 3). Some analogies made by the participants regarding their disease and symptoms are given below.

"You go into the sea, you dive into the water and can’t breathe… It is a kind of such an atmosphere. It will pull you down. You will be crippled by the bends. It is like a whirlpool…” (P11)

It is like a monster. A monster that takes everyone away…” (P18)

#### Subtheme 3.2. Analogies for nurses

Participants made similar and sometimes different definitions for nurses, focusing on their self-care status. Participants mostly simulated an "angel" for nurses. The majority were positive analogies, and two were described as "beings in hell" (Table 3). Some of the participants' statements are as follows:

"Nurses tried to save our lives by ignoring their own lives…”(P6)

"Nurses do not say anything offensive. They cheer you…” (P9)

“You are welcomed with the service and attention you do not have in your home…” (P15)

### Theme 4. Attitudes towards the care provided

The evaluations of individuals can contribute to the effectiveness and regulation of the services they receive. In this theme, most participants stated that they were "satisfied" with the care services offered to them. In this theme, there was a need to determine a subtheme and Maslow's hierarchy of human needs was taken into account when determining subthemes (Table 3). The subthemes and sample expressions created for this theme are given below.

### Respiration

Seventeen participants stated that they had respiratory distress, 10 of them had difficulty breathing, and seven expressed their respiratory distress in their analogies of COVID-19. Five patients with respiratory distress were followed up with a continuous positive airway pressure (CPAP) mask. Three of these five patients stated that they did not want to take CPAP and two stated that wearing a CPAP mask relieved them. Some of the participants' statements are as follows:

“The nurse put me in a machine to expand my lungs (CPAP mask). This machine is locking down. I could not cough on that machine and I was so clogged. When I could not, I was suffocating cough in that machine…” (P13).

“They put me in a machine, a big headgear (CPAP mask). I had not been able to sleep for 14 days, I slept for two days in that mask. The mask comforted me like I was born of my mother. They putt hat mask until I was back to normal. “Daughter, put on a helmet,” I would say. They would put it on, then they would take it off…” (P14).

### Nutrition

Most participants stated that they had problems with nutrition. Some of the participants' statements are as follows:

“Since I could not eat, the nurses fed me with a syringe…” (P11)

“My nurse even made me eat. He brought something and prepared it with his own hands…”(P23)

Excretion and privacy

In the case of being cared, 16 of the participants expressed their problems in terms of excretion and privacy. Of the participants, 12 expressed that they were embarrassed/hesitant when changing their sheets and diapers, 11 were uncomfortable unclothed, and nine had difficulty using diapers and were not able to go to the toilet. Some of the participants' statements are as follows:

“I did not know there was such a thing on earth until then. I was amazed by what you did when we were lazy to change our own child's diapers. A man changes a woman's diaper, a woman changes a man's diaper, and they do not make you feel it. I always say that this is not a job to be done with money…” (P10).

“I was so embarrassed when I changed my sheets and diapers. I say, “My consciousness is clear; I cannot accept it, why are you wearing diapers on me? ”Oh, my God, they stripped it, they put it in the machine.” (P17).

“According to the intensive care conditions, nothing should be worn. The room makes you feel bad. I wish something could be arranged for the patients. I think that patients who are conscious can feel better then…”(P23).

### Sleep

Eight participants stated that they experienced insomnia problems in the intensive care. One participant stated that the CPAP mask facilitated his/her sleep. Some of the participants' statements are as follows:

“I'm about to go to sleep, they come at night and take X-rays…” (P6)

“Sometimes there could be an excessive noise. The laughs and speeches of the nurses could create noise…” (P21)

### Communication

Most participants stated that the approaches of healthcare professionals positively affected them. Fourteen participants emphasized the importance of communication support of their relatives and healthcare professionals in coping with the negative emotions they experienced. Some of the participants' statements are as follows:

“Nurses and doctors showed a lot of interest. I have seen a lot of attention. The nurses treated me like a human being. The nurses comforted me by saying that I am the most comfortable patient here…”(P4).

“I was relieved when the nurse showed me the video of my husband’s well-being. When I talked to my niece on the phone, I was relieved to hear their voices…” (P18)

## Discussion

### Theme 1. Emotional expressions regarding the COVID-19 intensive care experience

In this study, the positive experiences of the participants regarding the intensive care unit were mostly focused on the interest shown to them. Negative statements and experiences were mostly determined to be respiratory distress, fear of death, nutritional distress and insomnia. Studies have shown similar results to the results of this study. Respiratory distress caused by COVID-19 can affect psychotic conditions that may develop in the patient and trigger death anxiety^14,15^. In their study, Zaybak et al.^16^ found that among the intensive care stressors experienced by patients, the primary ones were "pain", "fear of death" and "hearing the sounds that indicate heart problems from heart monitoring". Hintistan et al.^17^ found that intensive care patients had many bad experiences. Disease is a bad experience in itself. In addition to this experience, a sense of helplessness can accompany it. The intensive care unit, which is equipped with devices that make complex and strange noises, does not allow sunlight to enter and you can see every movement, which can cause anxiety. Poor experiences in an intensive care setting can only be minimized by the quality of care provided to patients.

### Theme 2. Coping methods

In this study, the participants stated that they coped with the negative emotions they experienced during the intensive care process with the support of healthcare professionals and their families. These results are consistent with the literature. Sahoo et al. (2020) stated in their qualitative study that a patient tried to reduce his anxiety and concerns by praying^18^. In a study, people who experienced COVID-19 turned to religion as a coping strategy^19^. In a study on religiosity during the pandemic, there was a 50% increase in Google searches on prayer-related issues compared to before the COVID-19 pandemic^20^**.**

Belief systems are the most frequently used method in the fight against disease as well as many problems. Spiritual care strengthens the immune system and enables patients to cope with stress and shorten the disease process^1^. Situations encountered by the patients and determination of the methods of coping with the disease can be determinant in the support and service systems to be offered to the patients. Psychological support, drug support and spiritual support for those who request them are primary ones that come to mind in this sense.

### Theme 3. Analogies

There may be some situations that are difficult to explain in daily life. In these cases, thoughts can be expressed more easily by comparing one concept to another^21^. Therefore, in this study, participants were asked to make a metaphor to describe COVID-19 and nurses.

For COVID-19, the participants used analogies such as "flu", "monster", "a stopper in the liver", "wolf in me", "tornado", "death", "haul", "Grim Reaper", and "bag in the head". As can be understood from these analogies, the worst experience of COVID-19 patients is not to be able to breathe.

The participants were asked to make an analogy to better understand what they thought about the nurses who spent the most time with them during their stay in intensive care units. As a result, the participants made analogies such as "angel", "family member" and "ladybug" for the nurses. One participant stated negatively with the definition of "sergeant" and the other with the definition of "being in hell". Positive descriptions are often heard in simulations in daily life. In the studies conducted, nurses mostly described themselves as "mothers", "superhero", and "saviors"^22,23^. Negative descriptions may be due to a disruption or nonfulfillment of something desired.

Individual evaluations are made with the effect of emotional states or reactive approaches that occur in instant situations. In fact, both negative analogies belong to the participants who have experienced intense physical restraint and panic attacks. Some participants stated that nurses sometimes respond late to patient requests. This may be associated with the nurses' workload, lack of staff due to the pandemic and problems with time. There are studies showing that nurses who care for intensive care patients are worried about getting sick or being a carrier and have dilemmas about their practices in intensive care units^24–27^. The problems experienced by nurses can also negatively affect nursing care. For this reason, the psychosocial health of nurses should be assessed and hospital management should provide support systems.

### Theme 4. Attitudes towards the care provided

Considering the participants’ experiences on the care provided, the titles of respiration, nutrition, excretion and privacy, sleep and communication come to the fore. Swallowing and respiratory distress cause malnutrition in COVID-19. Stopping oral intake during treatment may cause malnutrition. Malnutrition, which affects mortality and morbidity rates, is seen in between 30 and 40% of intensive care patients^28–30^. A participant stated that he/she lost 25 kg in this study, which supports this situation. Sixteen participants stated that they had problems with nutrition for different reasons. It has already been demonstrated that malnutrition delays healing times and increases hospitalization periods. Therefore, the prevention, diagnosis, and treatment of malnutrition must be regularly included in the management of hospitalized COVID-19 patients in a rehabilitation department, to improve both short and long-term prognosis^31,32^. Studies clearly show that people have problems with nutrition, whether they are diagnosed with COVID-19 or not^33–35^.

In the present study, the participants stated that they were ashamed of having their clothes removed during the diaper change and due to their presence in the intensive care unit. Intensive care patients may not be able to protect their privacy for cognitive and physical reasons. It is the moral responsibility of healthcare professionals to protect the privacy of these patients. Health professionals should behave in the best interests of patients who cannot make decisions about privacy. If the patient's privacy is given due attention, the negative effects of the stressor on the disease can be reduced by eliminating the patient's concerns^36^.

Sleep quality is important for maintaining a healthy life and cell regeneration and shortening the duration of the disease due to its recovery. Insomnia directly affects the prognosis of the disease^37^. Normal sleep patterns and habits of the individual change completely in the intensive care. Intensive care units are clinics where a healthy sleep rhythm cannot be achieved due to their physical structure as well as routine and emergency applications to patients. In addition to arranging the environment to allow patients to sleep, meeting the habits of individuals even at a minimum level (for example, wearing a thin hospital shirt or underwear) can facilitate falling asleep. A participant said, *“As per the intensive care conditions, you should not wear anything. The room makes you feel bad. I wish something could be arranged for the patients. I think that patients who are conscious can feel better then…”(P23)*. Tanrıkulu et al. (2022) reported that a patient expressed insomnia problems in a case they examined^38^.

Communication in intensive care is one of the most basic nursing skills. Patients with intubation or tracheostomy have difficulty in communication. Communication problems can cause stress, fear and panic in patients. Trust-based communication with the patient can help reduce the patient's anxiety, confusion, and anxiety. Studies have shown that nurses do not communicate adequately with unconscious patients^39–41^. In a study, Ashworth showed that nurses lacked conveying subjects such as time, day and date, which are the simplest examples of communication, to patients^42^. In this study, patients stated that nurses communicated with them and mostly used positive expressions to cheer them up. This difference shows that the holistic approach in the nursing profession has reached a preferable level. Some of the participants stated that they were concerned about their relatives. Seven participants stated that they could not see their relatives during the period when they were hospitalized in the intensive care unit, while the others stated that they had the opportunity to see their relatives from a distance or from the glass partition by taking protective measures. Those who saw their relatives stated that their negative emotions decreased and their strength to fight against the intensive care environment increased. In general, there have been visitor restrictions in many clinics and intensive care units to reduce the risk of transmission of COVID-19. In a study, communication through phone and video calls was approved due to patient and visitor restrictions during the COVID-19 pandemic^43^. It is important to provide family-centered holistic care to accelerate the recovery of patients^44^. Being able to see their relatives for a few minutes or hear their voices can eliminate the feeling of loneliness that can reduce the patients’ anxiety. Therefore, it is useful to review the visit and interview procedures in the intensive care unit. Although individualized practices are on the agenda, the principle of justice should be applied in patient visits as in every area and every patient should have the right to be visited. On the other side, visiting a patient hospitalized in the intensive care unit can be unsettling and frightening for the relatives of the patient due to the risk of transmission. However, it should not be forgotten that the relatives of the patient take the risk of visiting despite all kinds of risks due to the fear of losing the patient without seeing or even touching him/her for the last time. Restrictions set for protection may cause lifelong regrets. In fact, the positive statements of the participants who were visited confirm this idea.

### Limitations

In this study, we interviewed patients discharged from the COVID-19 intensive care unit. However, there are some limitations to this study as well. The results cannot be generalized since the study was conducted in a single region. Another limitation is that we asked the patients about their past experiences after they were discharged. Retrospective assessment may not be 100% accurate as time intervenes. There is also a limitation in this manuscript with the degree of depth that has been reached in the analysis that has been merely descriptive.

## Results and recommendations

As a result, the participants of the present study experienced negative emotions such as fear of death/anxiety, sadness, loneliness, and helplessness during their intensive care stay. They mostly stated that they tried to cope with it through prayer and communication. They likened COVID-19 with deadly and respiratory-inhibiting tools and diseases. In this process, they made positive analogies (such as angels and family members) about the nurses who spent the most time with them. The participants stated that they had difficulties in breathing, nutritional discharge and privacy, sleep and communication related to the care provided.

In line with these results, it is possible to say from the statements of the participants that the recipient of the service in the intensive care experienced a more troublesome and difficult process than the service provider. For this reason, intensive care workers should consider the physiological and psychosocial needs of patients and provide in-service trainings for institutions on this subject.

## Data Availability

The data sets used and/or analyzed during the current study are available from the corresponding author on reasonable request.

## References

[1] Roman NV, Mthembu TG, Hoosen M (2020). Spiritual care‘A deeper immunity’—A response to Covid-19 pandemic. Afr. J. Primary Health Care Family Med..

[2] Jalili M, Niroomand M, Hadavand F, Zeinali K, Fotouhi A (2021). Burnout among healthcare professionals during the COVID-19 pandemic: A cross-sectional study. Int. Arch. Occup. Environ. Health.

[3] Pakpour AH, Griffiths MD (2020). The fear of COVID-19 and its role in preventive behaviors. J. Concurr. Disord..

[4] von Vogelsang AC, Göransson KE, Falk AC, Nymark C (2021). Missed nursing care during the COVID-19 pandemic: A comparative observational study. J. Nurs. Manag..

[5] Sert G, Torun S, Karatas M, Gozderesi Y, Okuyaz Ş, Yalçın SO, Yıldırım G, Nazik S (2021). Induviduals living with cancer in Turkey and the Covid-19 pandemic. ActaBioethica.

[6] Alan H, Tiryaki Şen H, Bilgin O, Polat Ş (2021). Alarm fatigue questionnaire: Turkish validity and reliability study. Istanbul Gelisim Univ. J. Health Sci..

[7] Chand S, Kapoor S, Orsi D, Fazzari MJ, Tanner TG, Umeh GC, Islam M, Dicpinigaitis PV (2020). COVID-19-associated critical illness—Report of the first 300 patients admitted to intensive care units at a New York City Medical Center. J. Intens. Care Med..

[8] Davydow DS (2010). The burden of adverse mental health outcomes in critical illness survivors. Crit. Care.

[9] Kiekkas P, Diamanto A (2011). Pyschiatric long-term complications of intensive care unit survivors. Crit. Care Med..

[10] Williams TA, Ho KM, Dobb GJ, Finn JC, Knuiman M, Webb SAR (2010). Effect of length of stay in intensive care unit on hospital and long-term mortality of critically ill adult patients. Br. J. Anaesth..

[11] Labrague LJ, de Los Santos JAA, Fronda DC (2022). Factors associated with missed nursing care and nurse-assessed quality of care during the COVID-19 pandemic. J. Nurs. Manage..

[12] Alankaya N, Kurnaz F (2022). Evaluation of ıllness perceptions of patients hospitalized with the diagnosis of COVID-19 in a pandemic hospital. Hacettepe Univ. Faculty Health Sci. Nurs. J..

[13] Liu H, Li X, Chen Q, Li Y, Xie C, Ye M, Huang J (2020). Illness perception, mood state and disease-related knowledge level of COVID-19 family clusters, Hunan, China. Brain Behav. Immun..

[14] Çuvadar Y, Çuvadar A (2021). COVID-19-related intensive care delirium: Risk factors, prevention and treatment policies. J. Health Acad..

[15] Rahşan Ç, Şahin B (2018). Experiences and anxiety-depression states of patients hospitalized in intensive care units. J. Nurs. Sci..

[16] Zaybak A, Çevik K (2016). Perception of stressors in the intensive care unit by patients and nurses. J. Med. Surg. Intens. Care Med..

[17] Hintistan S, Nural N, Öztürk H (2009). Experience of patients hospitalized in the intensive care unit. J. Intens. Care Nurs..

[18] Sahoo S, Mehra A, Suri V, Malhotra P, Yaddanapudi LN, Puri GD, Grover S (2020). Lived experiences of COVID-19 intensive care unit survivors. Indian J. Psychol. Med..

[19] Molteni F, Ladini R, Biolcati F, Chiesi A, Maria G, Sani D, Guglielmi S, Maraffi M, Pedrazzani A, Segatti P, Vezzoni C (2021). Searching for comfort in religion: insecurity and religious behavior during the COVID-19 pandemic in Italy. Eur. Soc..

[20] Bentzen JS (2021). In crisis, we pray: Religiosity and the COVID-19 pandemic. J. Econ. Behav. Organ..

[21] Erol Y, Özdemir T (2017). Metaphorical perceptions of prospective teachers receiving pedagogical formation training regarding pedagogical formation. Cumhuriyet Int. Educ. J..

[22] Kale E, Çiçek Ü (2015). Metaphor perceptions of nurses regarding their own professions. J. Health Nurs. Manag..

[23] Saldamlı A, Andsoy II (2021). Metaphors of surgical unit nurses for surgical nursing. J. Acad. Res. Nurs..

[24] Entertainment R, Simsek N (2014). Evaluation of nurses' knowledge about spirituality and spiritual care. Acıbadem Univ. J. Health Sci..

[25] Kürü A (2022). A qualitative study on the causes and consequences of nurses' emotional labor behaviors in the Covid-19 pandemic. Business.

[26] Lake E, Narva AM, Holland S, Smith J, Cramer E, Rosenbaum K, French R, Clark R, Rogowski J (2022). Hospital nurses’ moral distress and mental health during Covid-19. J. Adv. Nurs..

[27] Silverman HJ, Kheirbek RE, Moscou-Jackson G, Day J (2021). Moral distress in nurses caring for patients with Covid-19. Nurs. Ethics.

[28] Bayır H, Yıldız İ, Erkurtaran MK, Koçoğlu H (2015). Malnutrition in intensive care patients. Abant Med. J..

[29] Giner M, Laviano A, Meguid MM, Gleason JR (1996). In 1995, there was a correlation between malnutrition and poor outcome in critically ill patients. Nutrition.

[30] Huang YC, Yen CE, Cheng CH, Jih KS, Kan MN (2000). Nutritional status of mechanically ventilated critically ill patients: Comparison of different types of nutritional support. Clinical Nutr..

[31] Barazzoni R, Bischoff SC, Breda J, Wickramasinghe K, Krznaric Z, Nitzan D, Pirlich M, Singer P (2020). ESPEN expert statements and practical guidance for nutritional management of individuals with SARS-CoV-2 infection. Clin. Nutr..

[32] Volkert D, Beck AM, Cederholm T, Cruz-Jentoft A, Goisser S, Hooper L, Kiesswetter E, Maggio M, Raynaud-Simon A, Sieber CC, Sobotka L (2019). ESPEN guideline on clinical nutrition and hydration in geriatrics. Clin. Nutr..

[33] Habiba L, Belahsen R (2023). Health problems associated to nutrition and lifestyle changes in the COVID-19 era. Bioact. Compd. Health Dis..

[34] Manca R, Bombillar F, Glomski C, Pica A (2022). Obesity and immune system impairment: A global problem during the COVID-19 pandemic. Int. J. Risk Saf. Med..

[35] Alan S, Gokyildiz Surucu S, Avcibay Vurgec B, Cevik A (2021). An investigation of individuals' health anxiety during the COVID‐19 pandemic within the framework of the functional health patterns. Perspect. Psychiatr. Care.

[36] Mersin S, Kahraman BB (2019). Respect and privacy for the dignity of the intensive care patient and his family. J. Intens. Care Nurs..

[37] Uslu Y, Korkmaz FD (2015). Sleep in intensive care patients: Nursing care. J. Educ. Res. Nurs..

[38] Tanrıkulu F, Erol F, Gündoğdu H, Koç F, Dıkmen Y (2022). Nursing care of Covid-19 patient receiving treatment in the intensive care unit: Case report. İzmir KâtipÇelebi Univ. J. Faculty Health Sci..

[39] Alasad J, Ahmad M (2005). Communication with critically ill patients. J. Adv. Nurs..

[40] Baker C, Melbey V (1996). An investigation ito the attitudes and practices of intensive care nurses toward verbal communication with unconscious patients. J. Clin. Nurs..

[41] Efil S, Founder NM, Eser O (2011). The effect of the frequency of visits to the relatives of the patients followed up in the neurosurgery intensive care unit and the communication with the nurse on the recovery of the patient. Kocatepe J. Med..

[42] Ashworth P (1981). Communicating with patients and relatives in the intensive care unit. Communication In Nursing Care.

[43] Kennedy NR, Steinberg A, Arnold RM, Doshi AA, White DB, DeLair W, Nigra K, Elmer J (2021). Perspectives on telephone and video communication in the intensive care unit during COVID-19. Ann. Am. Thorac. Soc..

[44] Aktaş Y, Arabacı LB (2016). Communication with the patient and his family in the intensive care unit. İzmir Kâtip Çelebi Univ. J. Faculty Health Sci..

